# Effects and mechanisms of acupuncture analgesia mediated by afferent nerves in acupoint microenvironments

**DOI:** 10.3389/fnins.2023.1239839

**Published:** 2024-02-07

**Authors:** Zezhi Fan, Baomin Dou, Jiangshan Wang, Yongjian Wu, Simin Du, Jiashan Li, Kaifang Yao, Yanwei Li, Shenjun Wang, Yinan Gong, Yi Guo, Zhifang Xu

**Affiliations:** ^1^Research Center of Experimental Acupuncture Science, Tianjin University of Traditional Chinese Medicine, Tianjin, China; ^2^School of Acupuncture and Moxibustion and Tuina, Tianjin University of Traditional Chinese Medicine, Tianjin, China; ^3^National Clinical Research Center for Chinese Medicine Acupuncture and Moxibustion, Tianjin, China; ^4^Tianjin Key Laboratory of Modern Chinese Medicine Theory of Innovation and Application, Tianjin, China

**Keywords:** acupuncture, acupoint, afferent fibers, analgesia, acupuncture effect

## Abstract

In the past few decades, the use of acupuncture analgesia in clinical practice has increased worldwide. This is due to its various benefits, including natural alleviation of pain without causing various adverse effects associated with non-steroidal anti-inflammatory drugs (NSAID) and opioids. The acupoint represents the initial site of acupuncture stimulation, where diverse types of nerve fibers located at the acupoint hold significant roles in the generation and transmission of acupuncture-related information. In this study, we analyzed the patterns and mechanisms of acupuncture analgesic mediated by acupoint afferent fibers, and found that acupuncture stimulates acupoints which rapidly and directly induces activation of high-density primary afferent fibers under the acupoints, including myelinated A fibers and unmyelinated C fibers. During acupuncture stimulation at the muscle layer, the analgesic effects can be induced by stimulation of A fiber threshold intensity. At the skin layer, the analgesic effects can only be produced by stimulation of C fiber threshold intensity. Electroacupuncture (EA) activates A fibers, while manual acupuncture (MA) activates both A and C fibers. Furthermore, acupuncture alters acupoint microenvironments, which positively modulates afferent fibers, enhancing the transmission of analgesic signals. In addition to local activation and conduction at acupoints, nerve fibers mediate the transmission of acupuncture information to pain centers. In the spinal cord, acupuncture activates neurons by inducing afferent fiber depolarization, modulating pain gating, inhibiting long-term potentiation (LTP) of the spinal dorsal horn and wide dynamic range (WDR) neuronal activities. At higher nerve centers, acupuncture inhibits neuronal activation in pain-related brain regions. In summary, acupuncture inhibits pain signal transmission at peripheral and central systems by activating different patterns of afferent fibers located on various layers of acupoints. This study provides ideas for enhancing the precise application and clinical translation of acupuncture.

## 1 Introduction

Acupuncture, a form of Chinese traditional medicine, involves insertion of a needle into “acupoints” for treatment. It can improve and prevent pain recurrence. The World Health Organization (WHO) has expanded the range of diseases that acupuncture can treat to 91 diseases, including multiple pains, and 16 other diseases. A previous bibliometric analysis revealed that many clinical trials have confirmed that acupuncture has clear effects on pain in different parts, and as an alternative for opioid drugs, it is widely used to treat cancer-related postoperative pain (Lee I. S. et al., 2020). A meta-analysis involving 17 randomized controlled trials (RCTs) (a total of 1,111 patients) published in 2019 by JAMA Oncology, a top international medical journal, showed that compared with the sham operation control group, acupuncture and acupoint pressing significantly reduced the pain intensity for cancer patients (He Y. et al., 2020). Another systematic study involving 13 clinical RCTs and about 1,000 patients revealed that acupuncture could significantly alleviate symptoms such as pain, morning stiffness, swelling of patients with rheumatoid arthritis and improve their quality of life (Seca et al., 2019). Liang et al. investigated the long-term efficacy of acupuncture in the treatment of migraines in JAMA Internal Medicine. In the 16-week RCT study, they found that acupuncture exerted persistent effects for at least 24 weeks in treatment of migraines, and there were no acupuncture-related serious adverse reactions (Zhao et al., 2017). Acupuncture elicits specific endogenous effects by modulating internal homeostasis, making it an effective alternative to exogenous pharmaceuticals which have side effects and are limited by occurrence of drug resistance (Chen et al., 2022).

Acupoints are the initial response site for acupuncture stimulation and the key sites for the transformation, amplification and transmission of acupuncture information. Compared with non-acupoint tissues, most acupoint tissues have denser nerve distribution (Langevin and Yandow, 2002; Li et al., 2004). Application of acupuncture to acupoints elicits a rapid and direct activation of peripheral sensory nerves (Kagitani et al., 2005). Ma et al. found that prokineticin receptor 2, the sensory neuron in *Zusanli* (ST36), mediates the anti-inflammatory effects of acupuncture via the vagus–adrenal pathway, providing the neuroanatomical basis underlying the effects of acupoints on autonomic nerve pathway. Excitation of afferent nerves located in acupoints represents a critical mechanism for initiating and propagating acupuncture effects (Qin et al., 2010; Kim et al., 2017; Liu et al., 2021). The *deqi* sensation, induced by acupuncture and local moxibustion-like stimulation, is more likely to be mediated by multiple afferent fibers simultaneously (Su et al., 2014). For instance, injection of 2% lidocaine hydrochloride at ST36 or cut the peripheral nerve, blocks signal transmission from local nerves to the central nervous system by inhibiting afferent fibers, thereby suppressing the effects of acupuncture (Huang et al., 2009; Uchida et al., 2010). Selective disruption of C fibers within acupoint regions through methods such as severing or capsaicin administration can inhibit acupuncture analgesic effects (Tjen-A-Looi et al., 2005). Nonetheless, the introduction of type I collagenase to acupoints to disrupt connective tissue did not have any influence on these acupuncture effects, as reported previously (Yam et al., 2018). Therefore, it shows that the activation of local afferent fibers at acupoints contributes to the generation of acupuncture-related information. In this study, we reviewed studies investigating acupuncture analgesia mediated by afferent fibers in the past 20 years, and summarized the mechanisms underlying the analgesic effects of acupuncture via afferent fibers. In so-doing, we provide a new basis for investigating the clinical application of acupuncture to treat various pain diseases.

## 2 Methods

### 2.1 Search strategy

Using the PubMed and Web of Science database, we retrieved studies published from 2003 to 2023. The search keywords employed were as follows: (“acupuncture” or “TEAS”) and (“A fiber” or “C fiber” or “afferent fiber” or “nociception”) and (“pain” or “analgesia”). The retrieved studies included basic research, reviews, clinical trials, and original research. A total of 715 articles were identified.

### 2.2 Study selection

**Figure 1:**
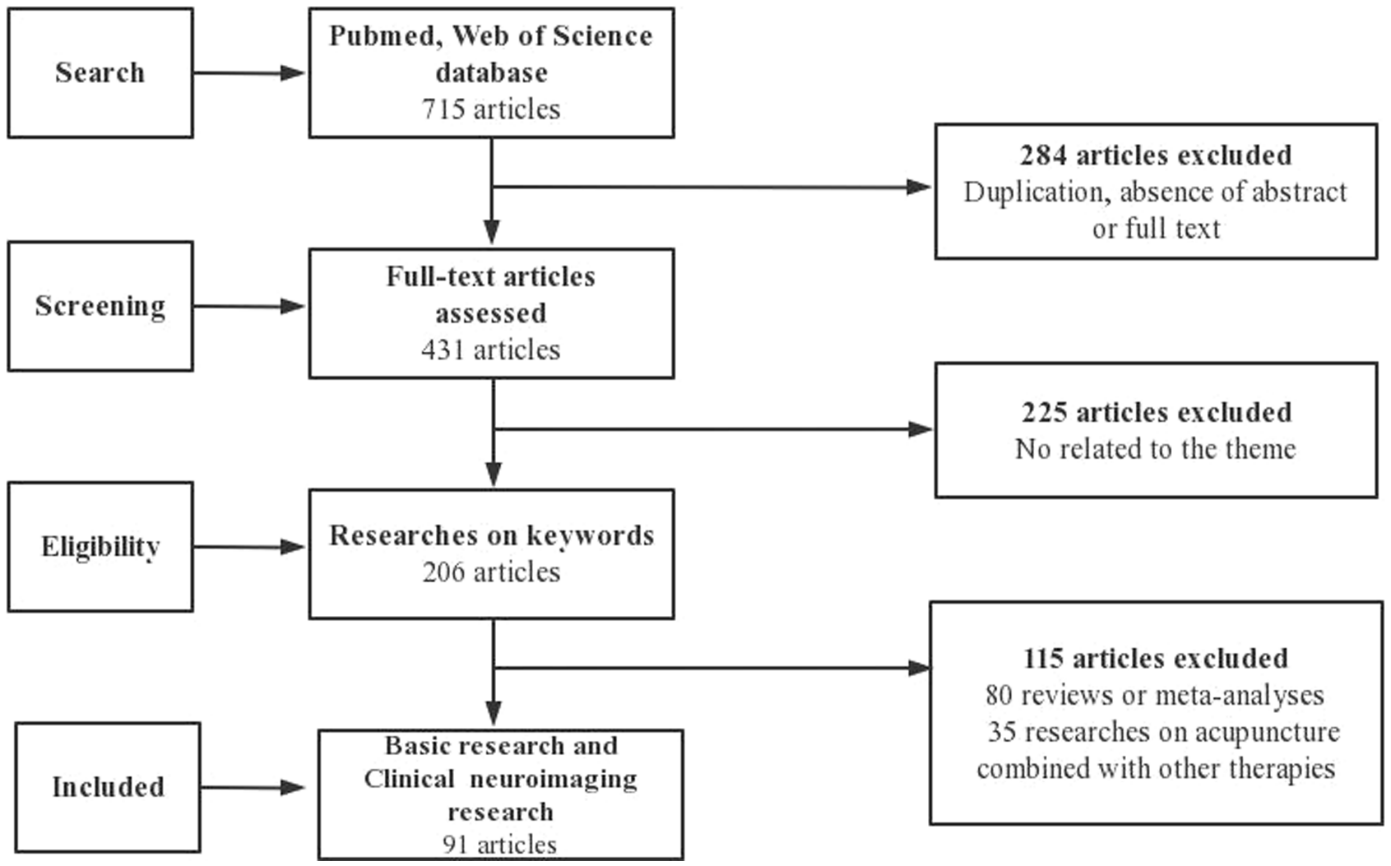
Flow chart of the search strategy and process.

The following inclusion criteria were used for screening of the selected articles written in English: stimulation methods included acupuncture, EA and TEAS, and main content is related to analgesia via afferent fibers. To ensure the inclusion of only relevant studies in the analysis, we employed manual selection using Excel software, followed by a comprehensive examination of their complete texts. We excluded the following studies from the analysis: 284 articles due to duplication, lack of abstracts or complete texts; 225 articles unrelated to our research topic; 80 articles that were either reviews or meta-analyses; and 35 articles that incorporated other therapies. As a result, 91 full-text basic research papers met the inclusion criteria (Figure 1).

To address the potential overlap among studies, we extracted important data of afferent fibers and acupuncture analgesia from a select group of published works. In the data extraction process, we employed a predetermined table that delineated the model type, intervention particulars (encompassing details about the acupuncture technique, acupoint selection, and acupuncture parameters), as well as the outcome measures. One author performed the initial data extraction, which was then reviewed by the other authors. The key information extracted from the 20 recent representative studies is provided in Table 1.

**Table 1:** Regulatory effects of acupuncture on afferent fibers activity.

References	Model	Intervention methods	Acupoints	Acupuncture parameters	Effect measurements	Afferent fibers activity regulated by acupuncture
Lee J. H. et al. (2020)	Oxaliplatin injection	BV	ST36	Subcutaneous injection	Cold and mechanical allodynia ↑	Current Threshold for Action Potential Generation in A Fiber DRG Neurons ↑
Gao et al. (2015)	Diarrhea or constipation	MA	LI11, ST37, BL25, ST25	2 Hz, rotated right then left about 180°, 60 s	Colonic motility ↑	Activation of C fiber ↑
Qin et al. (2014)	Diarrhea or constipation	MA	LI11, ST37, BL25, ST25	2 Hz, rotated right then left about 180°, 60 s	Jejunal motility ↑, Intrajejunal pressure ↓, Constipation or diarrhea frequency↓	Activation of C fiber ↑
Hino et al. (2010)	Irritated bladder	Sacral acupuncture	Periosteum of the sacral segment	Rotated manually 1.0–1.5 turns per second for 1 min turns per second for 1 min	Intercontraction interval ↑	Capsaicin-sensitive C fiber activation ↓
Xin et al. (2016)	ASIC3 gene knockout TRPV1 gene knockout	EA	ST36	50 Hz, 20 min	Mechanical pain thresholds ↓, Thermal pain thresholds ↓	After gene knockout, intensity on C-fiber reflex ↓
Fan et al. (2021)	Hypertension	MA	PC6, SI5	20 Hz at 1 min-intervals, 10 min	WDR ↑, Single fiber recordings ↑	Sensitivity of both somatic A and C fibers ↑
Guo et al. (2018)	Gastric distension	MA, EA	ST36, PC5, PC6, BL40	MA 2 Hz, EA 2 Hz 0.3–0.5 mA, 0.5 ms duration	Von Frey hairs threshold ↓	After TRPV1 blockade afferent fiber activity↓
Duanmu et al. (2020)	CFA	EA, TENS	ST34	2 Hz/100 Hz, 1 ms wave width, 30 min, 3 days	Abnormal electromyography ↓	In thresholds of A and C, abnormal electromyography ↓
Chen et al. (2021)	CFA	MA, EA	BL57	MA at a depth of 2 mm for 10 min; EA 10 Hz, 0.2 ms, 1 mA	Difference score of weight-bearing ↑	Muscular A fibers activity ↑ in deep EA Cutaneous C fibers activity ↑ in MA
Song et al. (2021)	CFA	MA	ST36, BL60	2 r/s, 4 r/s, 1 min, 7 days	Paw withdrawal latency ↑	Activates of C fibers ↑ in 4 r/s MA
Duan-Mu et al. (2021)	CFA	EA	ST34	2/100 Hz, 30 min	LTM Neurons ↑, WDR Neurons ↓	Spontaneous firing of LTM neurons ↑ WDR neurons ↓ by EA with intensity of threshold of A fibers
Xue et al. (2020)	Spared nerve injury	EA	ST36, SP6	2 Hz, 0.6 ms pulse width 10 min, 14 days	TrκB mRNA ↓, Mechanical hypersensitivity ↓	C fiber-evoked discharges of WDR neurons in the spinal dorsal horn ↓
Qu et al. (2020)	Acute migraine	EA	GB8, GB2	0.5 ~ 1 mA, 2 ~ 15 Hz	Mechanical pain threshold ↓, WDR neuronal spikes ↓	C fiber evoked WDR neuronal discharges of the trigeminocervical complex ↓
Ma et al. (2009)	Neuropathic pain	EA	GB30, BL40	1 Hz, 30 mA, 7 min, 2 days	Mechanical pain threshold ↑	C fiber high-frequency-stimulation induced LTP ↓
Li et al. (2019)	Postherpetic neuralgia	EA	GB30, GB34	1 mA, 30 min	Netrin-1↓, UNC5H2 ↑, Mechanical thresholds ↑	Resiniferatoxin-induced myelinated primary afferent nerve fiber sprouting into the spinal cord lamina II ↓
Wu et al. (2013)	Postherpetic neuralgia	EA	GB30, GB34	1 mA and 0.1 ms, 2 and 15 Hz, once every other day	Paw withdrawal thresholds ↑	Myelinated afferent nerves and their abnormal sprouting into the spinal lamina II ↓
Dai et al. (2019)	Incision pain	EA	SP6, GB34	Pulse width: 0.2 ms, intensities ranging 1–2-3 mA and each intensity for 10 min	Mechanical allodynia ↓	LTP of C fiber-evoked potentials in the spinal cord ↓
Peng et al. (2019)	Normal female	TENS	Radial nerve	100 Hz, 4 Hz TENS, 30 min	Alpha electroencephalography ↑	Aδ and C cutaneous nociceptors ↑ by low-frequency and high-intensity TENS
Liu et al. (2011)	Visceral pain	EA	ST2, GB14	2 Hz, 20 Hz, 1–5 mA	Abdominal contractions↓, C-fos expressions in NTS↓, in PTN↑	Aδ and C fibers c-fos expression ↑ in NTS
Li Y. Q. et al. (2007)	Normal rat	EA	ST13, LI11, CV6, ST21, ST36, ST21, BL21	0.8 and 2 thresholds for the activation of Aδ fiber, 1.5 threshold for the activation of C fiber, 30 s	Gastric motility response ↑ in Aδ fiber reflex threshold, ↓ in C fiber reflex threshold	Activation of Aδ afferent fibers ↑ in Aδ fiber reflex threshold Activation of C afferent fibers ↑ in C fiber reflex threshold

## 3 The role and rule of afferent fibers in signal initiation and conduction at acupoints induced by acupuncture

### 3.1 The role of afferent fibers in signal initiation and conduction at acupoints

At acupoints, there is a dense presence of primary afferent fibers, consisting of various myelinated A fibers, including medium-diameter Aδ fibers and larger-diameter Aβ fibers, as well as unmyelinated C fibers. The majority of Aδ and C fibers end in the superficial layers (laminae I and II) of the spinal cord, whereas Aβ fibers extend to deeper laminae III to V (Yam et al., 2018).

Sensory nerves, also referred to as “scouts” of the nervous system, play a crucial role in detecting acupuncture signals, forming generator potentials, encoding various afferent impulses, initiating neuromodulation, and mediating the generation as well as transduction of acupuncture information (Li et al., 2004). A recent study revealed that a complex of Schwann cells and unmyelinated C nerve endings can respond to mechanical stimuli in the cutaneous epidermis and translate them into electrical signals (Abdo et al., 2019). Bee venom acupuncture has been shown to decrease the pain signal transmission to the spinal cord by increasing the lowered action potential threshold induced by chemotherapy drug treatment in A fiber dorsal root ganglion (DRG) neurons (Lee J. H. et al., 2020). Meanwhile, C fibers also play pivotal role in generation of acupuncture effects (Qin et al., 2014; Gao et al., 2015). In a bladder stimulation model, sacral acupuncture significantly reduced acetic acid-induced acute inflammatory bladder pain and considerably prolonged the urinary intervals. However, when rats were pretreated with capsaicin to induce desensitization of C fibers, no noticeable effects of sacral acupuncture were observed in the experimental group (Hino et al., 2010). EA at *Jianshi* (PC5) and *Neiguan* (PC6) inhibited pressor reflex in rats with gastric dilatation. After removal of C fibers by capsaicin, the rats were no longer sensitive to the inhibitory effects of EA (Tjen-A-Looi et al., 2005).

The physical signals generated by the mechanical stimulation of acupuncture cannot be transmitted directly in the organism, but must be transduced into neuroelectrical or biochemical information through mechanical transduction in order to be recognized and transmitted by the organism. Mechanosensitive ion channels (MSCs) are activated by mechanical forces to regulate cellular functions and downstream signaling pathways. Studies have demonstrated that the body may sense the mechanical force of acupuncture through a variety of MSCs present in the acupoint area, and trigger the mechanotransduction process of acupuncture through corresponding ionic changes and intracellular signaling. Acupoints express a variety of receptors that are sensitive to pain, itch, temperature, mechanical stimulation and tissue damage (Guo et al., 2022). These include mechanosensitive ion channels, such as the transient receptor potential channel receptor (TRP) family and acid-sensing ion channel 3 (ASIC3). ASIC3 known to mediate acid and mechanical responsiveness located mainly in Aβ fiber innervating the skin and muscle, TRPV1 ion channel is highly expressed in sensory Aδ and C fibers. And one study found that ASIC3 knockout mice had a reduced analgesic effect in response to local 0.3 mA intensity EA, while TRPV1 knockout mice had a reduced analgesic effect in response to distal 1.0 mA intensity EA. These findings suggest that segmental analgesic effects of low-intensity EA can be mediated by ASIC 3 receptors on Aβ fibers, whereas the systemic analgesic effects of high-intensity EA can be mediated by Aδ and C fibers on TRPV1 receptors (Xin et al., 2016).

Acupuncture is a type of benign microinvasive stimulation that involves inserting acupoints and inducing the deformation of local connective tissues, leading to release of various chemical substances, such as interleukins, chemokines, tumor necrosis factors, adenosine triphosphate (ATP) and high migration molecules (Goldman et al., 2010; Zhang et al., 2018). These molecules are secreted into the extracellular space and bind their corresponding receptors, which results in the recruitment of mast cells, fibroblasts, neutrophils, and monocytes/macrophages, thereby promoting signal cascade amplification of acupuncture and activating the nuclear factor kappa-B (NF-κB), mitogen-activated protein kinase (MAPK), extracellular regulated protein kinases (ERK), and other signaling pathways in cells (Park et al., 2014; Zhang et al., 2021). These effects enhance intercellular communication as well as information transmission, and improve network connectivity of local microenvironments of acupoints (Wu et al., 2015). The ATP produced by acupoint microenvironments can activate afferent fibers via the P2X receptors, such as the ligand-gated ion channel 3 receptor (P2X3) on sensory nerves after acupuncture. When ATP breaks down into adenosine, it can activate the A1 receptor, which has analgesic effects. Injecting A1 receptor agonist at ST36 reduces the perception of sciatic nerve injury in the anterior cingulate cortex. This happens by activating adenosine A1 receptors on unmyelinated C fibers and Aδ fibers in the superficial peroneal nerve (Goldman et al., 2010). Therefore, the chemicals released from the acupoint microenvironment after acupuncture acting on nerve endings receptors may be an important mode of the analgesic effect of acupuncture (He J. R. et al., 2020). Upon activation by acupuncture, C fibers release substance P (SP) and CGRP, which engage in reciprocal interactions with the surrounding immune cells, thereby initiating local immune modulation (Wu et al., 2015; Talagas et al., 2020; Lowy et al., 2021). In hypertensive rat models, the increase in SP levels released from afferent nerve endings under acupoints on the wrist enhanced the sensitivity of A and C fibers to acupuncture stimulation, significantly enhancing signal transduction to achieve acupuncture effects (Fan et al., 2021). There was a higher presence of subepidermal nerve fibers expressing TRPV1 in the acupoint compared to non-acupoint regions. Moreover, the application of EA stimulation in acupoints notably enhanced the expression of TRPV1 in these nerve fibers. Furthermore, TRPV1 found in Aδ and C afferent nerves actively participates in the acupuncture-induced reduction of reflexive blood pressure elevation. This underscores its critical role in transmitting acupuncture signals from the peripheral to the central nervous system (Abraham et al., 2011; Guo et al., 2018).

In summary, sensory fibers that transmit acupuncture stimulation at acupoints can mediate the effects of acupuncture. Alterations in acupoint microenvironments after acupuncture also exert positive regulatory effects on incoming fibers.

### 3.2 Analgesic effects of acupuncture on afferent fibers are associated with layer of stimulation

Following inflammation of the hindpaw, myelinated, CGRP-positive neurons projecting to the paw skin displayed elevated mechanical currents in response to mechanical stimuli. Conversely, muscle inflammation markedly amplified mechanical currents in myelinated, CGRP-negative neurons projecting to muscle. These data suggest that mechanically gated currents are amplified in vivo following tissue inflammation (Weyer et al., 2015). After acupuncture, there may be inflammatory responses at the local acupoint, which vary in intensity based on the specific skin and muscle layer targeted by acupuncture. This differential stimulation activates distinct neural fibers, exerting analgesic effects. Both TEAS at the threshold intensity of C fibers and EA at *Liangqiu* (ST34) acupoint with A fiber threshold intensity within the muscle layer demonstrate pain-alleviating effects and normalize abnormal myoelectric discharge in rats with Complete Freund adjuvant (CFA) -induced inflammatory pain (Duanmu et al., 2020). Applications of EA targeting the muscle layers at the *Chengshan* (BL57) acupoint has been found to alleviate chronic gastrocnemius inflammation. However, when cobra snake venom is used to degrade the myelin sheath blocking the A fibers and obstruct the muscular A fibers input, the analgesic effects of EA were alleviated. Even after cutaneous nerve resection, the effects of deep EA analgesia were not alleviated. Hence, muscular A fibers play a crucial role in mediating EA-induced analgesia (Chen et al., 2021).

### 3.3 The type of activation of afferent fibers by acupuncture is associated with stimulation mode and intensity

Low current strength EA is sufficient to excite Aβ and Aδ fibers, which are often associated with pain and numbness. All afferent fibers (Aβ, Aδ, and C) are activated by MA, which manifests by heaviness and swelling of deep tissues below the acupoints (Kagitani et al., 2010). The C fibers, which transmit pain-associated noxious stimuli, are activated in greater numbers with increasing stimulus intensity. In CFA-induced inflammation pain rat models, MA at ST36 with 4 r/s activated more C fiber neurons than with 2 r/s, producing better pain relief. Therefore, MA may inhibit pain by inducing pain (Song et al., 2021). In a study involving CFA rats treated with EA at ST34 acupoint, EA at the A fiber threshold intensity significantly inhibited abnormal electromyography spontaneous activities induced by inflammation and relieved inflammatory pain (Duan-Mu et al., 2021). In addition, Aβ and Aδ fibers can also act as filters to prevent high-frequency signals from entering the central nervous system (CNS), and EA treatment at frequencies above 250 Hz are ineffective in relieving pain (Kagitani et al., 2010).

### 3.4 Analgesic effects of afferent fibers in acupoint sensitized states

Under pathological stress conditions, the relevant acupoints undergo various changes, including a reduction in pain thresholds. This change from a “silent” to an “active” state is referred to as “acupoint sensitization” and is an important indicator for acupoint selection. In rat models of knee osteoarthritis shows that increased hyperpolarization-activated current density in C-type neurons of L5 DRG mediates *Dubi* (ST35) acupoint sensitization (Zhang et al., 2019). It has also been found that some DRG neurons innervate both acupoints and pain sites. Using a chemogenetic, tracer-based approach, Li et al. found that some DRG neurons expressing the nociceptive neuron marker TRPV1 innervate both the ST36 acupoint and the ipsilateral hind paw (IHP) plantar. Inhibition of these shared neurons induced analgesia in the CFA pain model and obstruction of nociceptive sensation in normal mice, and elevated the mechanical pain threshold of ST36 acupoint in the CFA model (Li et al., 2022). It has been hypothesized that heightened excitability of shared injury-responsive neurons, which innervate both lesions and acupoints, may represent a neural mechanism underlying acupoint sensitization, particularly at the primary afferent neuron level. However, it is improbable that the TRPV1-expressing shared DRG neurons play a role in the analgesic effects induced by acupuncture at ST36 acupoint, as these neurons actually facilitate pain perception in the hind paw. Several studies suggest that type A fibers, which include proprioceptive neurons, are more likely to receive stimulation at acupoints through acupuncture. Consequently, the neurons activated by acupuncture at acupoints for therapeutic effects may not be the same group of neurons involved in acupoint sensitization. We hypothesize that the gate theory, which posits that sensitized ST36 acupoints and IHP are located in the same spinal cord segment, might be one of the mechanisms underlying acupuncture analgesia. However, further research is needed to determine whether sensitized shared neurons in the DRG aid in enhancing the transmission of signals from ST36 acupuncture to the central nervous system, ultimately contributing to its analgesic effects.

In summary, the achievement of analgesia varies depending on the specific location of lesion, necessitating the use of different stimulation intensities. At the muscle layer, stimulation at A fiber threshold intensity produces analgesia, whereas at the skin layer, stimulation at the C fiber threshold intensity produces analgesia. In terms of stimulation method, EA mainly activates the A fibers, while manual acupuncture activates both A and C fibers. In clinical practice, the layer and intensity of acupuncture stimulation should be considered according to the location of the pain in order to improve efficacy and to select the appropriate stimulation technique.

## 4 Acupuncture activates afferent fibers at acupoints to produce central analgesia

The spinal dorsal horn acts as the primary receiver of acupuncture signals carried by peripheral afferent fibers through the DRG. After an initial stage of integration, these acupuncture signals are then conveyed to the brain via ascending pathways such as the spinothalamic tract. In addition, the spinal dorsal horn also receives signals originating from the descending pathways of the brainstem, which serve to suppress information originating from the painful site within the particular spinal cord segment. Acupuncture through multi-level and multi-channel, multiple targets to adjust a complex network of mediated between the peripheral and central analgesia effect, acupuncture analgesia essence can be understood as afferent fibers from the lesion area can carry the pain of acupuncture and acupoint carries information of different levels of integration process in the central nervous system. While the present study focuses on the acupuncture inhibiting focal and spinal pain messages to the higher center.

### 4.1 Acupuncture activates acupoint afferent fibers to mediate central analgesia in the spinal cord

The gate control theory suggests that afferents from both peripheral sensory A fibers and C fibers activate upstream transmitter cells (T-cells) in the posterior horn of the spinal cord and form synaptic connections with posterior horn layer II cells (SG-cells). When C fibers are excited, the gates open and cause pain responses; when A fibers are excited, the gates close and inhibit pain responses (Duan et al., 2014). When pain is produced, A and C fibers interact to inhibit and regulate the gates to maintain a dynamic balance between pain and analgesia, alleviating the body’s pain responses. Light touch is mediated predominantly by Aβ afferents with low mechanical thresholds. The sensation of painful touch is triggered by high-threshold C and Aδ nociceptors. This explains why gently touching a wound can alleviate pain. When TEAS or EA is applied to the painful area, it may effectively “close the gate” that transmits pain perception (Lumpkin and Caterina, 2007). This is achieved by activating A fibers, which work to inhibit the transmission of pain signals to the spinal cord via C fibers in the affected organs (Sluka and Walsh, 2003; Leung et al., 2005).

In the spinal cord, acupuncture activates neurons that are at rest to exert analgesic effects (Fink et al., 2004; Kagitani et al., 2005). Mechanistically, EA promotes spontaneous CFA inflammatory muscle pain-induced firing of C type low-threshold mechanoreceptor neurons in the dorsal horn of the spinal cord and inhibits the activities of various neurons (Duan-Mu et al., 2021). In sciatic selective nerve injury models, EA at ST36 and *Sanyinjiao* (SP6) reduced C fiber-induced firing of WDR neurons in the dorsal horn of the spinal cord, inhibiting neuronal excitability and improving pain hypersensitivity symptoms (Xue et al., 2020). The EA stimulation at *Shuaigu* (GB8), for 60 s produced analgesic effects in acute migraine, which were associated with acupuncture inhibiting C fiber-evoked WDR neuronal firing in dorsal horn neurons of the atlantoaxial spinal cord in the trigeminal cervical complex (Qu et al., 2020). Meanwhile, studies have shown that acupuncture can regulate the excitability of C fiber related neurons by modulating neurotransmitters in the dorsal horn portion of the spinal cord. It was found that LTP of C fiber-induced potentials in the spinal cord is a substrate for central sensitization of pain pathways, which amplifies injurious inputs and leads to nociceptive hyperalgesia (Sandkühler, 2007). In contrast, EA at *Huantiao* (GB30) and *Weizhong* (BL40) can inhibit central sensitization-induced abnormal LTP of dorsal horns of the spine and relieved neuropathic pain in nerve-injury rat models (Ma et al., 2009). Moreover, EA reduces abnormal sprouting of afferent nerves into the spinal: activation of μ-opioid receptors by EA suppressed nociceptive transmission induced by arbutin-mediated germination of myelinated afferent nerve fibers in the dorsal horns of the spinal cord by inhibiting protein expressions of Netrin-1 and its receptors (Li et al., 2019), can also suppress resiniferatoxin-induced tactile allodynia by attenuating the damage of myelinated afferent nerves and their abnormal sprouting into the spinal lamina II (Wu et al., 2013).

Acupuncture can also inhibit the release of neurotransmitters and active substances from the spinal cord by suppressing myelinated glial cell functions via ridges. Pretreatment and post-modeling treatments with EA at ST36 and BL40 acupoints significantly inhibited the activation of spinal microglia and neurons after formalin injection. Meanwhile, EA significantly suppressed the expression of interferon- γ, interleukin-6, SP, CGRP and increased interleukin-4 expressions, thereby reducing the formalin-induced injurious outcomes. Dai reported that EA at SP6 and *Yanglingquan* (GB34) increased interleukin-10 expression in spinal astrocytes, inhibited spinal longitudinal enhancement between primary afferent C fibers and dorsal horn neurons and reduced incisional pain (Clark et al., 2015; Dai et al., 2019).

### 4.2 Acupuncture activates afferent fibers to mediate brain advanced central analgesia

Transmission of acupuncture information from primary afferent fibers to the spinal dorsal horn is followed by its transmission via the superior conduction pathway to the brain, where it activates the downstream inhibitory system (Li A. et al., 2007). This activation modulates pain transmission and release of mediators with analgesic effects, such as 5-hydroxytryptamine and acetylcholine (Chen et al., 2020). Simultaneously, the acupuncture signals are conveyed to the brain, which is highly receptive to information from afferent fibers, particularly those associated with pain (Rudomin et al., 2004; Ritter et al., 2013; Vierck et al., 2013; Whitsel et al., 2019). The N-Methyl-D-Aspartate (NMDA) receptors amplify the C fibers to primary somatosensory cortex messaging in a frequency-dependent manner (Kalliomäki et al., 2003). A randomized controlled trial involving 80 human volunteers found that “acupuncture-like” TENS selectively activates Aδ and C skin injury receptors, and enhances the functional connection between primary motor and somatosensory cortex brain regions and medial prefrontal cortex brain regions (Peng et al., 2019). In a separate study, nociceptive EA stimulation targeted orofacial acupoints. This stimulation activated Aδ and C fibers, leading to increased neuronal excitability in the paratrigeminal nucleus (PTN). Additionally, it inhibited visceral pain and neuronal excitability in the nucleus of the solitary tract (NTS) in rats. When infraorbital nerves were transected or pre-treated with capsaicin, EA analgesia was hindered. However, pretreatment with snake venom had no impact on EA analgesia. These findings suggest that the PTN-NTS secondary neural pathway may play a role in EA analgesia, facilitated by the activation of small-diameter (Aδ and/or C) fibers (Liu et al., 2011).

### 4.3 Acupuncture induces spinal segmental analgesia via afferent fibers

Activation of afferent fibers by acupuncture is segment-specific, and the acupuncture effects produced vary based on stimulation thresholds of afferent fibers, which are determined by anatomical relationships between target organs and acupoints. At the same nerve segment on the side of the pain source, below the Aδ fiber activation threshold intensity of acupuncture can produce analgesic effect (Zhu et al., 2004). However, the analgesic effects of acupuncture in heterotopic nerve segments require the involvement of supraspinal centers. Ectopic EA stimulation below the Aδ fiber activation threshold is completely ineffective, EA stimulation intensity greater than the activated Aδ fiber stimulation intensity or even reaching the threshold of the C fiber stimulation intensity can produce analgesic (Xu et al., 2003; Kim et al., 2006; Liu et al., 2011). While the activation of A nerve fibers within the same or related segments demonstrates clear effectiveness in pain inhibition, achieving analgesia in heterogeneous nerve segments requires the participation of additional nerve fibers. Further investigation and enhancement are needed to fully understand and refine the underlying mechanism. Notably, that the regulation of gastric motility by acupuncture revealed that acupuncture stimulation far from the area of gastric innervation required the presence of intact vagus nerves and spinal cord for realization of acupuncture effects, while acupuncture stimulation of acupoints in the same segment as the area of gastric innervation required the presence of intact sympathetic nerves for realization of acupuncture effects. The close relationship between sympathetic and vagus nerves and afferent fibers has also been reported (Li Y. Q. et al., 2007; Xing et al., 2015; Hu et al., 2017; Yu et al., 2021; Cui et al., 2022; Ji et al., 2022; Ma et al., 2023). Whether the adoption of different acupuncture intensities based on segmental relationships between acupoints and lesions is associated with afferent fibers and autonomic nerves needs to be further investigated to determine this.

In conclusion, opening and closing of “gates” in lower centers are influenced by higher central control and downstream control systems, in addition to A and C fibers (Melzack and Wall, 1965). Acupuncture activates silent neurons at rest by depolarizing the afferent fibers, inhibiting LTP of the spinal dorsal horn and WDR neuronal activities, and reducing pain-related protein expressions to exert analgesic effects. At the spinal cord level, there is segmental specificity between the target organ and the acupuncture point. When both are at the same nerve segment, acupuncture intensity that activates A fibers produce significant analgesic effects. However, at heterogeneous nerve segments, stimulation intensity greater than that of activated A fibers threshold even up to C fiber is required to result in significant effects. Pain-related brain areas are highly receptive to information from afferent fibers, and the analgesic effect ceases after needling inhibits neuronal activation in pain-related brain areas and antagonizes the afferent fibers.

## 5 Discussion

Acupuncture analgesia is common in the field of acupuncture therapy. Studies have investigated the mechanisms of acupuncture analgesia from the aspects of peripheral receptors, types of afferent fibers, dorsal root ganglion responses, interactions of spinal dorsal horns, spinal ascending pathway, participation of the endogenous analgesic system, and integration of advanced central information to send descending inhibitory signals. There have been advances in cognition of acupuncture analgesia, which is associated with fewer adverse reactions, among other advantages. Therefore, research on acupuncture analgesia should aim to clarify the internal principles guiding the regulation of acupoints at local, distal and systemic levels.

Primary afferent fibers play a crucial role in initiating and transmitting acupuncture’s analgesic effects, making them key factors that influence the efficacy of acupuncture in treating pain-related conditions. The generation of pain involves the peripheral and central nervous system, and the mechanism of acupuncture analgesia also involves all levels of the peripheral and central nervous system. The extensive regulatory effects of acupuncture and its relationship to analgesia are closely intertwined. Acupuncture analgesia can be comprehended as the integration process of information transmitted by afferent fibers from the pain area and acupuncture-specific information conveyed by acupoints within various levels of the CNS. Numerous studies have shown that both A and C fibers of the afferent nerves participate in the transduction of acupuncture signals. Some studies explored the relationship between Aδ and C fibers. It was found that C fibers can modulate neighboring Aδ fibers by releasing CGRP in axon-to-axon communication (Eftekhari et al., 2013; Edvinsson et al., 2019). However, whether A and C fibers communicate with each other via interactions during pain generation and transmission requires further investigations. As a benign, microinvasive stimulus, acupuncture induces the release of multiple chemicals under the epidermis, activating supracellular signaling pathways that facilitate intercellular communication, transmit acupuncture effects, and enhance the network connectivity of the microenvironment at acupoints. Changes in the microenvironment of acupoint increase the sensitivity of afferent fibers to acupuncture, facilitating the triggering of acupuncture signals and the conduction of acupuncture signals from the periphery to the CNS. This positively modulates the afferent fibers and enhances the generation of acupuncture effect. Primary afferent fibers then transmit the acupuncture information to the spinal cord, activating neurons that are typically silent in the resting state. This process regulates the expression level of pain-related molecules, integrates and modulates pain information in the spinal cord segments, and transmits the acupuncture information to the brain area via spinal cord processing. It causes the suppression of neuronal expression in the relevant brain region during the state of pain, ultimately yielding a systemic analgesic effect. The considerable reduction in the efficacy of acupuncture analgesia following the antagonism of afferent fibers confirms that the activation of these fibers mediates the effectiveness of acupuncture analgesia.

The effectiveness of acupuncture in activating afferent fibers to alleviate pain is influenced by several factors. At the muscle layer, the stimulation of A fiber threshold intensity can produce analgesic effect, at the skin layer, TEAS with C fiber threshold intensity can produce analgesic effect. If TEAS with A fiber threshold intensity is used, the stimulation intensity may be too low to produce significant analgesic effect. Additionally, there exists segmental specificity between the pain areas and the acupoint at the spinal cord level. When the pain areas and the acupoint are located at the same nerve segment, the stimulation intensity targeting A fibers can yield a notable analgesic effect. Regarding the acupuncture intervention modality, the high-intensity stimulation of manual acupuncture activates both A and C fibers, whereas the low-frequency stimulation of EA predominantly activate A fibers. Currently, EA is widely used in the treatment of pain and other diseases, and the traditional MA has gradually transformed into EA and TEAS, among other types.

Many RCTs research have also confirmed that 2 Hz low-frequency stimulation is the necessary parameter of EA in the treatment of angina pectoris, migraine and other diseases. The findings of this study are in line with the summarized principles of basic research outlined in this paper. The results indicate that EA may possess advantages over manual acupuncture. However, further research is imperative to increase our understanding of the mechanisms responsible for this discernible divergence. TEAS of high frequency and low intensity can stimulate A nerve fibers and inhibit the afferent nociceptive information at the level of spinal cord, which plays a local analgesic effect. In comparison, TEAS of high intensity can stimulate the type C nerve fibers and activate the central descending inhibitory system to produce analgesic effect. However, it is challenging to effectively transform acupuncture into EA and TEAS. For example, traditional acupuncture is a mechanical stimulation, and the mechanism of EA and TEAS as electrical stimulation is not consistent in initiating acupuncture effect at acupoints. It is difficult for EA to achieve the sensation of *deqi* and *shouqi* as traditional acupuncture techniques, which also needs translational research. From the perspective of the gate theory, it has been demonstrated that the activation of A fibers at acupoints can effectively suppress the pain sensation resulting from abnormal activation of C fibers at the site of the lesion. Intense stimulation of the skin surrounding the lesion triggers the activation of C fiber afferents within the affected area, leading to the inhibition of nociceptive sensory afferents and the reduction of inflammatory factor release in deeper muscles. However, whether acupuncture can inhibit the abnormal activation of C fibers in the lesion by activating C fibers in the acupoint area need to be further investigated. Therefore, conducting a thorough analysis and comprehensive summary of analgesic patterns, crucial stimulus intensities, and modalities across various acupuncture techniques and diverse pain models is of significant importance. This effort can greatly contribute to the formulation of pain treatment guidelines and the development of regulatory protocols. Investigating animal models that closely resemble clinical disease states, employing commonly used acupoint combinations and stimulation parameters, and exploring the underlying targets of acupuncture analgesia are key areas of focus in the field of acupuncture translational medicine. In recent years, large-scale single-cell RNA sequencing has been used to analyze the subtypes of primary sensory neurons and their receptor mechanisms in the dorsal root ganglion. In the future, researchers should explore whether the anatomical differences between different acupoints can activate different afferent nerve fibers and produce different analgesic mechanisms. The types of peripheral receptors and afferent nerves activated by different acupuncture and moxibustion interventions can reveal the analgesic characteristics and mechanism of different nerve fiber types mediating various acupuncture stimulation methods, and to provide scientific experimental evidence and stimulation techniques (level and intensity) scheme for the development of new surface regulation models and precise analgesia with acupuncture. This offers additional avenues for the application of acupuncture and acupuncture combined with medicine to treat, manage and control pain.

**Figure 2:**
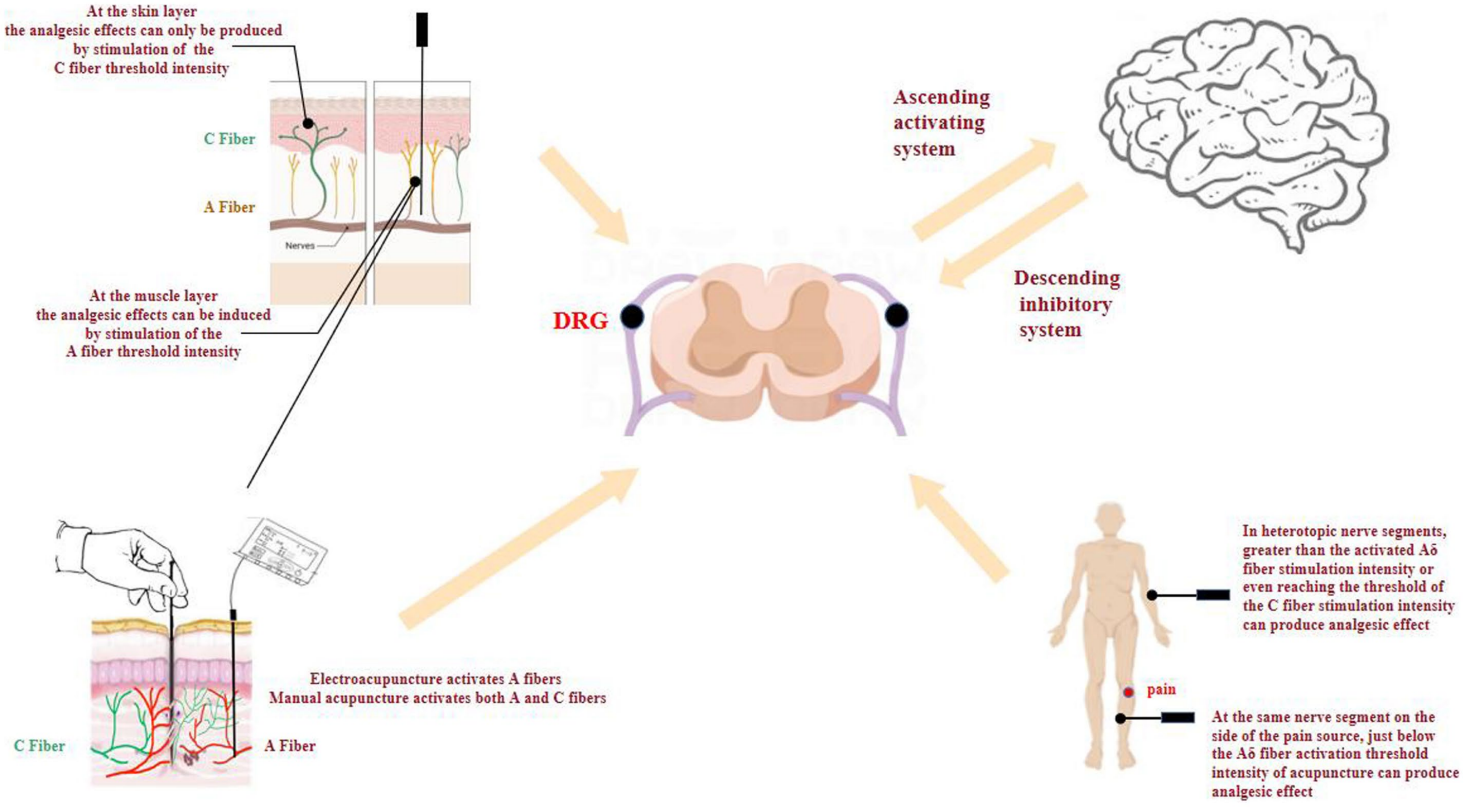
Acupuncture analgesia mediated by afferent fibers in acupoint microenvironment.

In summary, Figure 2 reveals that acupuncture and changes in the microenvironment of acupoints trigger the activation of afferent fibers. This activation enables the transmission of acupuncture-related information from the peripheral to the central nervous system. In addition, afferent fibers and acupuncture analgesia show some degree of regularity, in terms of acupuncture mode, intensity, layer, and segmental relationship between acupoints and lesions: (1) The analgesic effects of acupuncture differ depending on the layer of stimulation. When targeting the muscle layer, the analgesic response is mediated by the activation of A fibers at threshold intensity. Conversely, at the skin layer, the analgesic effect can only be elicited by reaching the threshold intensity necessary to activate C fibers; (2) EA with low-frequency stimulation volume mainly activates A fibers, while MA with high-intensity stimulation volume activates both A and C fibers; and (3) When the pain areas and acupoints coincide at the same nerve segment, the analgesic effect can be prominently achieved through the activation of A fibers using an optimal stimulation intensity. Conversely, if the pain areas and acupoints are located at different nerve segments, an analgesic effect can only be observed when applying a stimulation intensity surpassing the threshold required for A fiber activation. Therefore, it is crucial to comprehensively consider the source of pain in order to enhance efficacy and select the most suitable stimulation technique.

## Author contributions

YGu and ZX conceived the project. SD, KY, and JL analyzed the data. ZF, BD, and JW wrote the manuscript draft. YL and YW prepared the figures and the graphical abstract. All authors contributed to the article and approved the submitted version.
